# Genome-wide association study for multiple phenotype analysis

**DOI:** 10.1186/s12919-018-0135-8

**Published:** 2018-09-17

**Authors:** Xuan Deng, Biqi Wang, Virginia Fisher, Gina Peloso, Adrienne Cupples, Ching-Ti Liu

**Affiliations:** Department of Biostatistics, School of Public Health, Boston University, 801 Massachusetts Avenue 3rd Floor, Boston, MA 02118 USA

## Abstract

Genome-wide association studies often collect multiple phenotypes for complex diseases. Multivariate joint analyses have higher power to detect genetic variants compared with the marginal analysis of each phenotype and are also able to identify loci with pleiotropic effects. We extend the unified score-based association test to incorporate family structure, apply different approaches to analyze multiple traits in GAW20 real samples, and compare the results. Through simulation studies, we confirm that the Type I error rate of the pedigree-based unified score association test is appropriately controlled. In marginalanalysis of triglyceride levels, we found 1 subgenome-wide significant variant on chromosome 6. Joint analyses identified several suggestive genome-wide significant signals, with the pedigree-based unified score association test yielding the greatest number of significant results.

## Background

The increasing availability of high-density genomic data with thousands of samples enables the identification of single-nucleotide polymorphisms (SNPs) contributing to complex traits on a genome-wide scale. Research studies often collect data on multiple related phenotypes to better understand disease structure; however, genome-wide association studies (GWAS) commonly analyze each trait independently. For example, body mass index (BMI) and waist-to-hip ratio (WHR) are both proxy traits for obesity and commonly collected in an obesity-related study. The standard approach usually analyzes each phenotype separately and reports the corresponding findings of each analysis, ignoring the dependency among traits. Approaches considering joint analyses have been proposed to tackle multiple phenotypes. Yang and Wang [1] and Ott and Wang [2] described a number of approaches elaborately, including multivariate regression models, variable reduction methods such as principal component analysis, and canonical correlation analysis. However, there is no single approach that is uniformly the most powerful across all situations. The sum of squared score (SSU) test does not explicitly incorporate trait correlation, and multivariate analysis of variance (MANOVA) could fail to detect pleiotropy when a strong trait correlation exists and the traits have thesame direction of association [3]. Considered to be an optimally weighted combination of MANOVA and SSU, the unified score-based association test (USAT) by Ray et al. [3] may provide higher power, especially for detecting pleiotropy.

We aimed to study the performance of various approaches for jointly analyzing multiple phenotypes. We first reviewed existing methods. We then expanded USAT to related samples as a pedigree-based USAT (pUSAT). We found that the Type I error rate of pUSATwas well preserved through simulations. Finally, we analyzed GAW20real data using multiple phenotype methods and compared the results.

## Methods

Assume *K* correlated phenotypes *Y*_1_,…, *Y*_*K*_ in *N* individuals. Let ***Y***_*k*_ be the *N* × 1 vector of *k*^*th*^ phenotype and ***Y*** be the *N* × *K* matrix for all individuals. The test of interest is the association of a single variant with the *K* phenotypes. Suppose *G*_*i*_ is the genotype score (ie, count of the minor allele as 0, 1, or 2) for a SNP of interest *i*, and ***G*** is the *N* × 1 vector of genotypes for all individuals. Moreover, define ***C*** = (*c*_1_, …, *c*_*q*_) as the *N* × *q* matrix of a set of *q*-adjusted covariates for all samples.

### Marginal linear mixed model

The linear mixed model (LMM) is frequently used to account for the sample relatedness or the cryptic relatedness due to population structure. For a given SNP, the standard LMM is:

1 $$ {Y}_k=\alpha +G{\beta}_k+\boldsymbol{C}{\gamma}_k+{Q}_k+{\epsilon}_k, $$

where *α* refers to the overall mean of *k*^*th*^ phenotype, *β*_*k*_ is the regression coefficient representing the linear fixed genetic effect on the *k*^*th*^ phenotype, and *γ*_*k*_ is a *q* × 1 vector of fixed covariate effects on the *k*^*th*^ phenotype. *Q*_*k*_ and *ϵ*_*k*_ are random effect and error, respectively, assumed to follow normal distributions ${Q}_k\sim N\left(0,\boldsymbol{\Phi} {\sigma}_g^2\right)$ and ${\epsilon}_k\sim N\left(0,{\sigma}_e^2\boldsymbol{I}\right)$, where ${\sigma}_g^2$ and ${\sigma}_e^2$ are genetic and environmental components of variance, ***I*** is an *N* × *N* identity matrix and **Φ** is an *N* × *N* matrix of pairwise measures of genetic relatedness.To handle multiple phenotypes, the most intuitive and simplest approach is to implement marginal LMM to test each SNP against 1 phenotype at a time. For the *k*^*th*^ marginal model, the null hypothesis is that the given SNP is not associated with the *k*^*th*^ phenotype (*H*_0*k*_ : *β*_*k*_ = 0). The estimation of parameters can be obtained through the maximum likelihood estimator (MLE) or the restricted MLE (RMLE) [4], and test statistics are constructed thereafter. Because multiple tests are conducted for each SNP, a modification of local significance level should be used to control the overall Type I error, such as Bonferroni correction. The marginal LMM completely ignores the correlation among traits, possibly reducing power, especially in the case of highly correlated phenotypes.

### SSU test

The results from the marginal test can be combined to simultaneously test the association of a given SNP to the multiple phenotypes. Yang and Wang [1] extended Pan’s [5] test statistic for the association between multiple rare or common variants and a single phenotype, and developed the well-known approach of the SSU test. The SSU test statistic is:

2 $$ {S}_{sq}={T}^TT=\sum \limits_{k=1}^K{t}_k^2 $$

where *t*_*k*_ is the association statistic for the *k*^*th*^ phenotype with a given marker from a marginal model, for example, from eq. (1). The distribution of eq. (2) can be approximated as a scaled noncentral chi-squared distribution $\mathrm{a}{\chi}_d^2+b$ with

$$ \mathrm{a}=\frac{\sum_{k=1}^K{c}_k^3}{\sum_{k=1}^K{c}_k^2},\mathrm{b}={\sum}_{k=1}^K{c}_k-\frac{{\left({\sum}_{k=1}^K{c}_k^2\right)}^2}{\sum_{k=1}^K{c}_k^3},\mathrm{d}=\frac{{\left({\sum}_{k=1}^K{c}_k^2\right)}^3}{{\left({\sum}_{k=1}^K{c}_k^3\right)}^2} $$

where *c*_*k*_’s are the eigenvalues of the variance–covariance matrix Σ of *t*_*k*_ [6]. The SSU test is derived from marginal modelsand does not consider the correlation structure explicitly; therefore, the power is not highly affected by increasing the degree of dependency among the traits. However, the SSU test suffers from power loss with a small proportion of associated traits.

### Multivariate linear mixed model

MANOVA considers the trait correlation directly in the test statistics and corresponding distributions [7]. For family data, the multivariate LMM (mvLMM) has been developed as a compelling method for testing multiple phenotypes. An mvLMM for the association of *K* phenotypes and a given SNP is:

$$ \mathbf{Y}=\upalpha +\mathrm{G}{\boldsymbol{\beta}}^T+\boldsymbol{C}{\boldsymbol{\gamma}}_{\boldsymbol{k}}+\boldsymbol{Q}+\boldsymbol{\epsilon} $$

where ***β*** is a *K* × 1 vector of the SNP genetic effect sizes for the *K* phenotypes; ***γ***_***k***_ is an *q* × *K* matrix of the corresponding coefficients for the covariates; *Q* is an *N* × *K* matrix of random effects with MVN distribution ***Q***~*MVN*_*N* × *K*_(**0**, ***Φ***, ***V***_***g***_), where **Φ** is the row covariance matrix for relatedness, ***V***_***g***_ is the *K* × *K* column covariance matrix for the genetic variance component; ***ϵ*** is an *N* × *K* matrix of errors with ***ϵ~****MVN*_*N* × *K*_(**0**, ***I***_***N × N***_, ***V***_***e***_), where ***I***_***N × N***_ is the row covariance matrix, and ***V***_***e***_ is the *K* × *K* column covariance matrix of the environmental variance component. The null hypothesis of interest is that the SNP effect sizes for all phenotypes are zero: *H*_0_ : *β*_1_ = … = *β*_*K*_ = 0. These parameters can be estimated through either MLE or RMLE [8]. The mvLMM typically has good performance when a few of phenotypes are associated with a SNP, but lacks power with high correlations among traits and the genetic effect sizes of the traits are similar in magnitude and in same direction.

### USAT and pUSAT

The true genetic sizes and the direction of associations are usually unknown a priori and therefore one would not know which approach is the best for the study. Ray et al. [3] proposed the USAT approach, which combinesMANOVA and SSU. USAT takes the advantages of MANOVA and SSU while not requiring the prior knowledge of true effect sizes or correlations among traits. The method was originally designed for independent samples. Let *T*_*w*_ be the weighted statistic *T*_*w*_ = *wT*_*M*_ + (1 − *w*)*T*_*S*_, where *w* is a weight from 0 to 1, *T*_*M*_ is the MANOVA test statistic, and *T*_*s*_ is the SSU test statistic combining the marginal results. *T*_*M*_ and *T*_*S*_ are the statistics from the analyses assuming the independence among samples. Under the null, *T*_*w*_ is approximately a linear combination of chi-squared distributions and the *p* value *p*_*w*_ of *T*_*w*_ can be calculated using Liu et al. [6]. The optimal USAT test statistic is:

$$ {T}_{USAT}=\underset{0\le w\le 1}{\min }{p}_w $$

and *w* can be considered from a grid of {*w*_1_ = 0, *w*_2_ = 0.1, …, *w*_11_ = 1}.

Here, we expand their method to related samples. Specifically, we define the proposed pUSAT as *T*_*w*, *pUSAT*_ = *wT*_*mvLMM*_ + (1 − *w*)*T*_*s*, *LMM*_, where *T*_*mvLMM*_ is the mvLMM test statistic, and *T*_*s*, *LMM*_ is the SSU test statistic combining the marginal LMM results. In this way, the relatedness among study participants is taken into consideration in the test statistic. Then, the optimal pUSAT test statistic is defined as:

$$ {T}_{pUSAT}=\underset{0\le w\le 1}{\min }{p}_{w, pUSAT} $$

where *p*_*w*, *pUSAT*_ is the *p* value of *T*_*w*, *pUSAT*_. An approximated *p* value for *T*_*pUSAT*_ using numerical integration is [3]:

$$ {\displaystyle \begin{array}{c}{p}_{pUSAT}=\mathit{\Pr}\left({T}_{pUSAT}\le {t}_{pUSAT}\right)=1-\Pr \left({T}_{pUSAT}\ge {t}_{pUSAT}\right)\\ {}=1-\Pr \left({T}_{w_1}<{q}_{\mathrm{min}}\left({w}_1\right),\dots, {T}_{w_{11}}<{q}_{\mathrm{min}}\left({w}_{11}\right)\right)\\ {}\approx 1-\int {F}_{T_S}\left({\delta}_w(w)|x\right){f}_{T_M}(x) dx\end{array}} $$

where *t*_*pUSAT*_ is the observed value, *q*_*min*_(*w*_*b*_) is the (1- *t*_*pUSAT*_)th percentile of the distribution of ${T}_{w_b}$ for w = *w*_*b*_, ${F}_{T_S}\left(\bullet \right)$ is the cumulative distribution function of *T*_*S*, *LMM*_, ${\delta}_w(x)=\underset{w\in \left\{{w}_1,\dots, {w}_{11}\right\}}{\min}\frac{{\mathrm{q}}_{min}(w)- wx}{1-w}$ and ${f}_{T_M}\left(\bullet \right)$ is the probability density function of *T*_*mvLMM*_. The details of this calculation can be found in Ray et al. [3]. The pUSAT is an application-directed approach and does not require knowledge of the underlying association. Weights to mvLMM can change according to the SNP being tested. pUSAT may be powerful in detecting pleiotropy for a large number of traits with weak correlation or a few of highly correlated phenotypes.

### Phenotypic and genotypic data

GAW20 provides the dense genome-wide SNPs from the 821 pedigree-based individuals with triglyceride (TG) and high-density lipoprotein cholesterol (HDL-C) levels measured. We used the log-transformed average of pretreatment values at visits 1 and 2 of TG and HDL-Clevels and investigated the pleiotropic variants involved in blood lipids. The GAW20 data has been genotyped using the Affymetrix Genome-wide Human SNP Array 6.0. SNPs were excluded with a call rate < 95%, minor allele frequency < 5%, and failure of the Hardy-Weinberg equilibrium test (*p* value<10e-6), which results in a total of 587,358 variants. Individuals with more than 5% missing genotypes were also excluded from analysis.

## Results

### Simulation study

To evaluate Type I error rate of the proposed pUSAT approach, we conducted simulation studies considering 2 correlated phenotypes. The phenotype data were simulated from the following model:

3 $$ \mathrm{Y}\sim {\mathrm{MVN}}_2\left(\mathbf{0},\boldsymbol{\Phi}, {\boldsymbol{V}}_{\boldsymbol{g}}\right)+{\mathrm{MVN}}_2\left(\mathbf{0},{\mathbf{I}}_{\boldsymbol{N}\times \boldsymbol{N}},{\boldsymbol{V}}_{\boldsymbol{e}}\right) $$

where ***V***_***g***_ ***=*** *h*^2^**B**(ρ), ***V***_***e***_ **=** (1 − *h*^2^)**E** and *h*^2^ is the heritability varying from 0 to 1. For the genetic covariance matrix **B**(ρ) and the environmental covariance matrix **E**, we used acompound symmetry (CS) correlation structure with **B**(ρ)_*ij*_ ***=*** **E**_*ij*_ ***=*** ρ, where a single parameter ρ can control the model and define the correlation among the phenotypes. We used the kinship matrix **Φ** ofGAW20 data and considered *h*^2^ as 0.5%. The different correlation ρ ‘s (ρ = 0, 0.25, 0.5, 0.75) were assessed in the simulation. We evaluated the Type I error rate using 1000 null phenotype data setssimulated from eq. (3) and variants on chromosome 21 fromGAW20 individuals, with minor allele frequencyvarying from 0.052 to 0.500. TheType I error rate is well controlled for the pUSAT approach (Table 1) although slightly conservative.

**Table 1:** Estimated Type I errors of pUSAT for K = 2 phenotypes (α = 0.0)

Type I error	Correlation ρ			
	0	0.25	0.5	0.75
pUSAT	0.027	0.032	0.036	0.038

### Real data analysis

The Pearson correlation coefficient, ignoring the family structure, between TG and HDL-Clevels (on log-scale) in the data set, is − 0.45. The empirical genetic relatedness matrix was calculated before conducting analyses. Besides SSU, mvLMM, USAT, and pUSAT, the univariate analyses were also performed and shown. All statistical models were adjusted for age, sex, indicators of field center, and smoking status, and implemented in GEMMA (genome-wide efficient mixed-model analysis) [4, 8]. Table 2 lists the descriptive statistics for the variables used in the model.

**Table 2:** Descriptive statistics of variables in the analysis

		Men(*N* = 407)	Women(*N* = 414)	Total(*N* = 821)
Log of TG^a^		4.86 (0.59)	4.70 (0.56)	4.78 (0.58)
Log of HDL-C^a^		3.69 (0.23)	3.92 (0.26)	3.80 (0.27)
Age^a^		48.29 (15.93)	48.38 (15.84)	48.34 (15.87)
Field center^b^	Minnesota	206 (50.6%)	205 (49.5%)	411 (50%)
	Utah	201 (49.4%)	209 (50.5%)	410 (50%)
Smoking status^b^	Never Smoker	268 (65.8%)	298 (72.0%)	566 (68.9%)
	Past Smoker	106 (26.0%)	82 (19.8%)	188 (22.9%)
	Current Smoker	33 (8.1%)	34 (8.2%)	67 (8.2%)

^a^mean (SD); ^b^count (frequency)

**Fig. 1:**
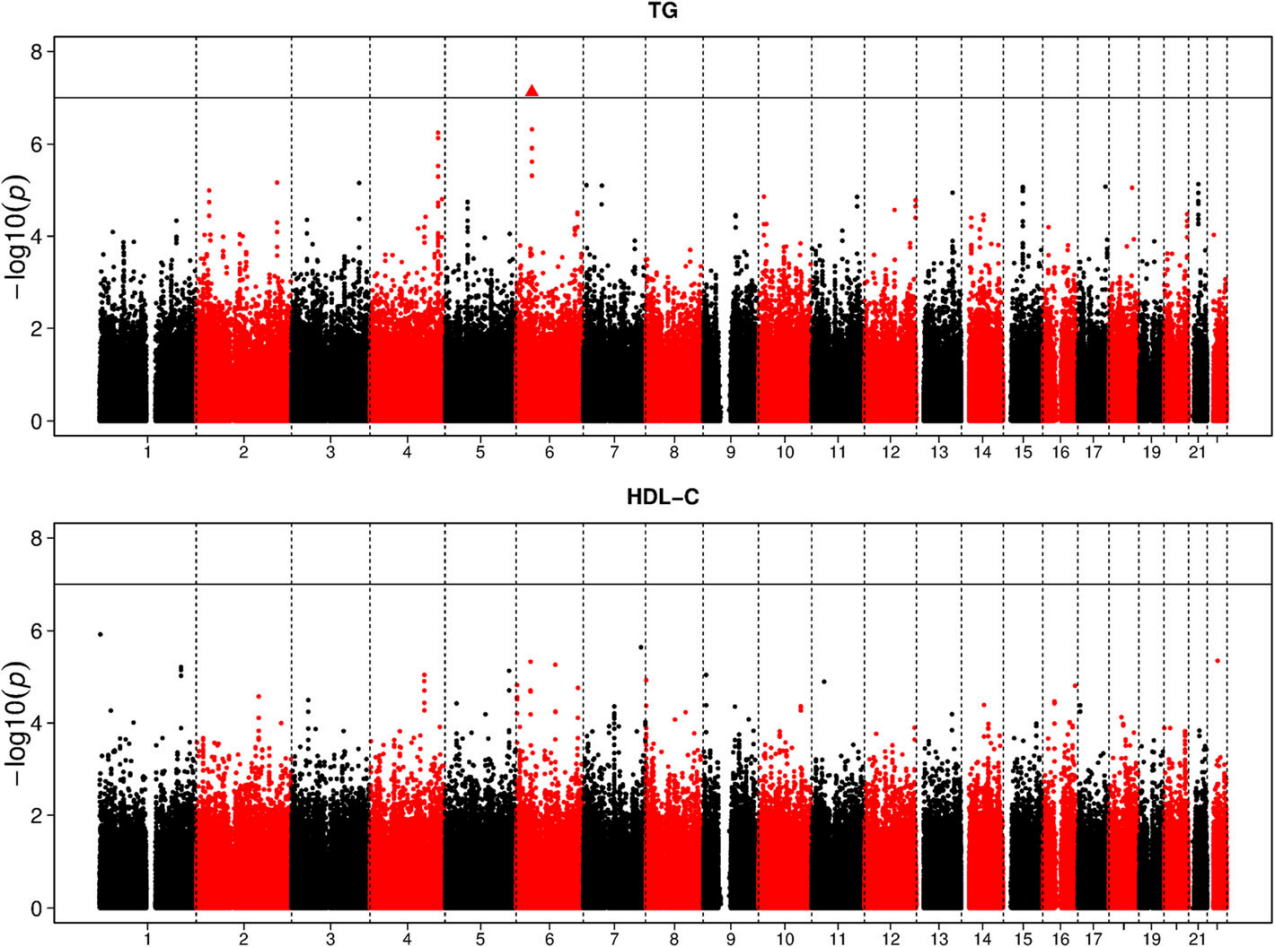
Manhattan plots of the univariateanalyses (TG and HDL-C) for chromosomes 1 to 22. The black horizontal line indicated the subgenome-wide significance threshold (*p* value = 1 × 10^− 7^)

**Fig. 2:**
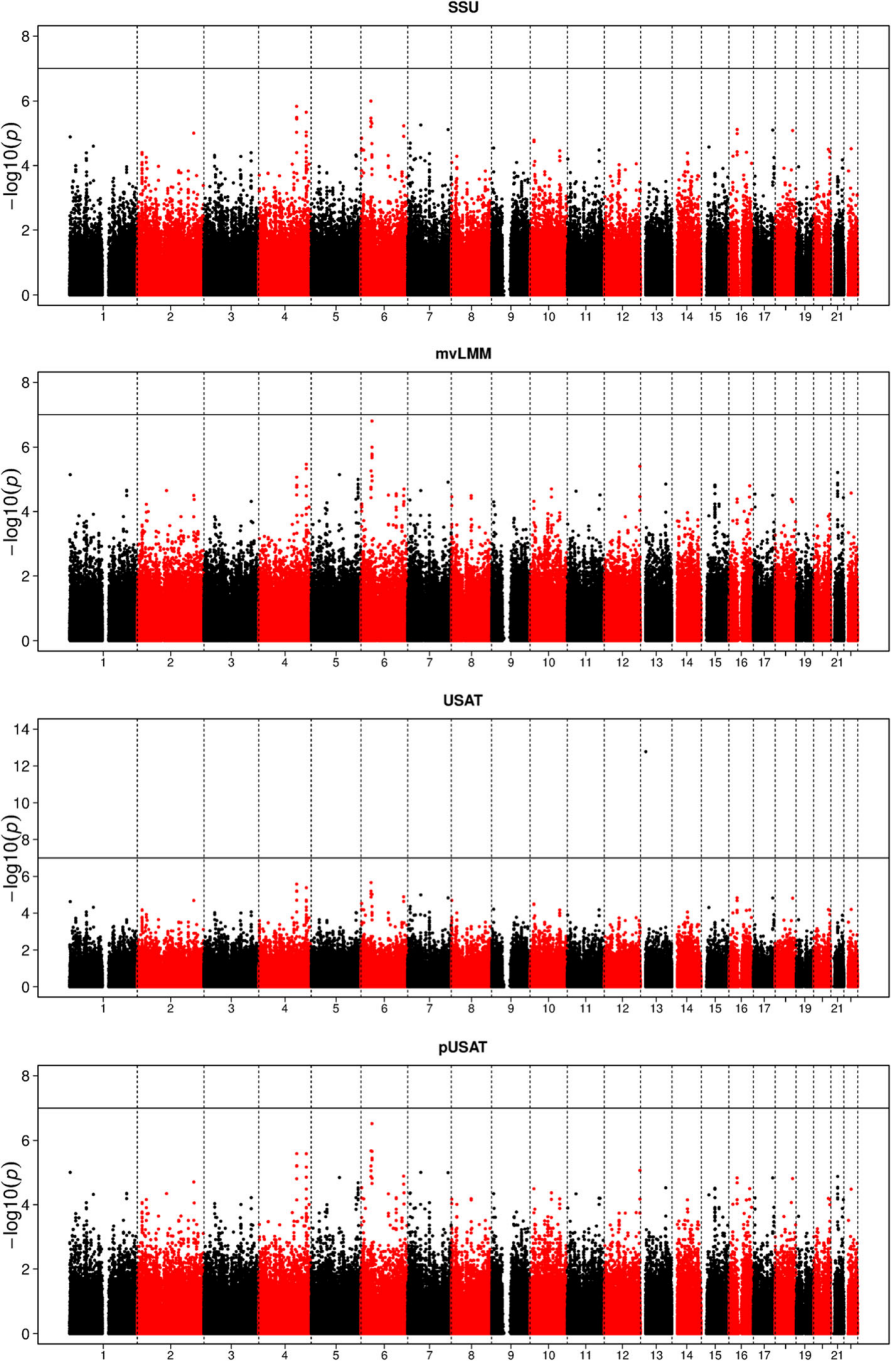
Manhattan plots of the multivariateanalyses (SSU, mvLMM, USAT, and pUSAT) for chromosomes 1 to 22. The black horizontal line indicated the subgenome-wide significance threshold (*p* value = 1 × 10^− 7^)

Figure 1 shows the Manhattan plots for the univariate analysis using marginal LMM. The horizontal line indicates the subgenome-wide significance level (*p* value = 1 × 10^− 7^). We observed no genome-wide significant SNPs for either phenotype; however, we did identify1 subgenome-wide significant locus on chromosome 6 (rs17619780 with a*p* valueof 7.2 × 10^− 8^ in gene *LRFN2*) as associated with TG levels. *LRFN2*is related to TGs in the suggestive genome-wide significance level (*p* < 5 × 10^− 6^) [9]; however, more exploration of this gene is needed. Interestingly, the heterozygous deletion of the *LRFN2* is reported to be associated with working memory deficits [10], a well-known complication of high TG levels. Furthermore, both USAT and pUSAT performed similarly (Table 3) except one genome-wide association (GWA)-significant variant, rs2880301, identified by the regular USAT. The reported *p* value of 1.69 × 10^− 13^ from USAT is suspicious as no other approach (neither univariate nor joint analysis) reports even nominal significance signal. In addition, USAT analysis ignored the dependency among the individuals, which could potentially lead to the inflated signals. In addition, we did not identify any signals that reached GWAsignificance by another multivariate approach (SSU, mvLMM, or pUSAT), as shown in Fig. 2. Some suggestive genome-wide significant signals were detected on chromosomes 4, 6, and 12. Most of the identified SNPs in Fig. 2 are in linkage disequilibrium, therefore, we kept 1SNP with the smallest *p* value as representative within ± 500 kb (Table 3). From Table 3, we found that the joint analysis is able to identify most variants that were significant in either marginal analysis and also catches 1 variant (rs7300117) missed in the univariate analysis, emphasizing the importance of multivariate joint analyses. pUSAT provides comparable results with slightly smaller *p* values by integrating information from SSU and mvLMM. Closer investigation shows that some SNPs are outstandingly noticeable for pUSAT, but not for SSUormvLMM, especially on chromosomes 2,3,4, and 6.

**Table 3:** SNPs that are suggestive as being of genome-wide significance (*p* < 5 × 10^− 6^) in univariate and joint analysis*

SNP	Chr:Pos	Univariate analysis (LMM)		Joint analysis			
		TG	HDL-C	SSU	mvLMM	USAT	pUSAT
rs90513	1:3189344	3.33E-02	**1.20E-06**	1.30E-05	7.18E-06	2.36E-05	9.88E-06
rs11940232	4:138953336	6.32E-05	1.98E-05	**1.47E-06**	8.56E-06	**2.65E-06**	**2.58E-06**
rs17058802	4:173880215	**5.66E-07**	4.56E-03	**2.23E-06**	**3.39E-06**	**4.13E-06**	**2.60E-06**
rs708010	6:37071350	1.86E-04	**4.69E-06**	**1.01E-06**	5.48E-06	**2.19E-06**	**2.12E-06**
rs17619780	6:40472303	**7.58E-08**	2.22E-01	**4.95E-06**	**1.59E-07**	9.60E-06	**3.01E-07**
rs12533593	7:147451966	6.69E-03	**2.28E-06**	7.72E-06	1.22E-05	1.48E-05	1.01E-05
rs7300117	12:130266575	2.24E-05	8.66E-01	5.60E-04	**3.92E-06**	9.99E-04	8.58E-06
rs2880301	13:18998534	9.66E-01	7.29E-02	1.95E-01	1.19E-01	**1.69E-13**	2.22E-01
rs17464499	22:26221715	3.20E-02	**4.48E-06**	3.02E-05	2.67E-05	6.30E-05	3.29E-05

*pValues of different approaches that reach suggestive genome-wide significance level are in bold

## Discussion and conclusions

The explosion in datacollection and the increasing evidence that some loci affect multiple traits require more complex statistical models for analyses to better understand the properties of association. Here, we reviewed several different methods for multiple phenotypes in GWAS, and expanded the USAT approach to related samples as pUSAT. The proposed method can provide insight into the underlying associations, and help the researchers to identify pleiotropic loci especially when prior information is unavailable. The simulation studies demonstrate that the Type I error rate of pUSAT is conservative under different correlations. We also applied various methods to the GAW20 data with TG and HDL-C as the phenotypes. One suspicious locus was identified as GWA-significant by the regular USAT, which assumes independent individuals, whereas other multivariate analyses missed this locus. Several suggestiveGWA loci were detected by the joint multivariate analyses; however, pUSAT highlights the importance of joint analysis for multiple phenotypes and yields smaller *p* values for most SNPs.
